# Study of Ultrasonic Near-Field Region in Ultrasonic Liquid-Level Monitoring System

**DOI:** 10.3390/mi11080763

**Published:** 2020-08-10

**Authors:** Wanjia Gao, Wenyi Liu, Yanjun Hu, Jun Wang

**Affiliations:** Key Laboratory of Instrumentation Science & Dynamic Measurement, Ministry of Education, North University of China, Taiyuan 030051, China; 18810577682@163.com (W.G.); 18234125986@163.com (Y.H.); 18834161512@163.com (J.W.)

**Keywords:** ultrasonic, liquid level measurement, echo energy, near-field region

## Abstract

In the method of monitoring the liquid level based on ultrasonic impedance, the near-field effect can seriously affect the validity of the results. In this paper, we explore the factors affecting the length of the ultrasonic near field. Based on that, we propose the optimal length and the minimum length of the buffer block to avoid the near field. The evaluations show that when the parameters of the ultrasonic probe are 15 mm in diameter, 1 MHz in frequency, and ±15 V in emitted ultrasonic wave amplitude, the best results are obtained when the length of the buffer block is 22 mm. When the probe diameter is 10 mm, the buffer block length should be ≥5 mm to ensure the validity of the measured results. The evaluation precision is 1 mm. This research can effectively avoid the blind area of emitted waves when using ultrasonic to measure the liquid level. It provides an effective basis for the selection and design of ultrasonic probes.

## 1. Introduction

In the field of aerospace, real-time monitoring and accurate measurement of liquid fuel consumption in fuel tanks are very necessary [1,2]. Therefore, the research and development of a liquid-level sensor are particularly important.

There are two types of liquid-level measurement technologies, which are invasive and non-invasive [3]. The invasive types include capacitive [4], resistive [5], float-type [6], magnetostriction type [7], optical fiber liquid-level meter [8], and many more. As the fuel tank is a closed container, its internal environment is high pressure, low temperature, etc., and its internal liquid fuel is inflammable and explosive. Therefore, it is not suitable to use a contact sensor introduced into the container to measure the liquid level [9,10]. Ultrasonic non destructive testing (NDT) technology has gradually become the mainstream for liquid-level detection [11,12].

There are some liquid-level measuring devices based on ultrasonic propagation characteristics, which are mainly divided into three categories: interface reflection method [13], penetrative method [14], and attenuation method [15]. The detection accuracy of the interface reflection method and the penetrative method is greatly affected by the temperature of the internal medium. For large containers with diameters over 1 m, the long transmission distance and bubbles or impurities in the liquid will seriously affect the transmission of ultrasonic waves. The penetration attenuation characteristics of liquid medium will also seriously affect the reliability of measurement [16,17]. Attenuation is a relatively new technique that requires only an ultrasonic transducer to be installed on one side of the container wall. When the internal medium at the measurement point is different, the attenuation range of ultrasonic echo energy on the container wall is different. According to the time from the reception of the echo to the attenuation, it can distinguish whether the internal liquid level reaches the detection point, so as to play the role of liquid-level monitoring [18,19]. Therefore, the ultrasonic attenuation method has relatively good measurement accuracy and reliability.

The ultrasonic transducer emits a beam of ultrasonic waves, but due to the existence of the near field, the effective reflection echo cannot be received, resulting in inaccuracy of the measurement. Therefore, when using ultrasonic waves for measurement, it is necessary to ensure that the measured surface is in the far-field area of the sound pressure to obtain an effective signal [20]. Buffer blocks are widely used in ultrasonic applications. At present, two kinds of rods with cylindrical and cone structures are used by researchers. Zhang et al. [21] studied the shape and boundary conditions of the buffer block and proposed a high-performance rod with shape based on a cone reference surface. Hoppe et al. [22] found an optimized geometry of a buffer rod for an ultrasonic density sensor. They can measure the amplitude with high accuracy and low noise. Fischer et al. [23] used a conical buffer element with a combination of two materials to obtain a reference for the pulse amplitude of the emitted signal. The buffer material connected to the transducer is polymethyl methacrylate (PMMA), and the material in contact with the measured liquid is high-grade steel. However, the acoustic impedance of the buffer block material is not close to that of the measured liquid, so the sensitivity is low. Liu et al. [24] made a detailed comparison description of the buffer block materials and drew the curve of the sound velocity in PMMA varying with frequency and temperature. Combined with other physical properties of PMMA, it is finally proposed that PMMA is most suitable for the measurement experiment of liquid acoustic properties.

To sum up, most of the researchers studied the material, shape, boundary conditions, and internal noise of the buffer block. For the length of the near-field area of ultrasound, the researchers only say that the acoustic beam range should be more than 3 times the length of the near field when using the p-wave testing [22]. However, if the length of the buffer block is too short, the near field region cannot be avoided, and if it is too long, it may cause ultrasonic attenuation. At present, no team has proposed an exact value of the optimal and the minimum size of the buffer block required to avoid the near-field area.

In conclusion, based on the attenuation method, this paper builds a fixed-point liquid-level monitoring system. This method is based on the ultrasonic impedance method: the ultrasonic transducer emits a group of continuous ultrasonic waves to monitor whether the height of the liquid level is higher than the transducer by measuring the energy values of the received echo of the container wall. In this paper, a buffer block is added between the probe and the container wall. We used different lengths of buffer blocks to conduct experiments and studied the relationship between the length of the near field of the ultrasonic wave and the amplitude of the received echo. Finally, the experiment was conducted to find the minimum size of the ultrasonic probe and buffer block that can get effective results when using this method for liquid-level monitoring. The research in this paper provides an effective solution to avoid the near-field area for experiments such as liquid-level measurement based on ultrasound. It also provides a powerful basis for the selection and design of ultrasonic probes in other experiments.

## 2. Theory and Methods 

### 2.1. Principle of Ultrasonic Impedance Method

This paper builds an experimental system for liquid-level monitoring based on the ultrasonic impedance method. Ultrasonic waves can propagate in any medium in the form of a wave. It propagates along a straight line [25]. In the process of transmission, diffraction, refraction, reflection, attenuation, and other phenomena will occur when encountering obstacles in the path [26]. When the ultrasonic transducer emits a beam of ultrasound and reaches the interface between the inner wall of the container and the internal medium, transmission and reflection will occur. The sound intensity reflectance, *R*, and sound intensity transmittance, *T,* can be calculated by Equations (1) and (2) [27]:

(1) $$R=\frac{I_{a}}{I}=\frac{{\left(Z_{2}-Z_{i}\right)}^{2}}{{\left(Z_{2}+Z_{i}\right)}^{2}}$$

(2) $$T=\frac{I_{t}}{I}=1-R=\frac{4Z_{2}Z_{i}}{{\left(Z_{2}+Z_{i}\right)}^{2}}$$

where $I_{a}$ is the reflected sound intensity, W/m^2^, $I_{t}$ is the transmitted sound intensity, W/m^2^, *I* is the incident sound intensity, W/m^2^, *Z*_2_ is the acoustic impedance of the tested container, Mrayl, and *Z*_i_ is the acoustic impedance of the internal medium, Mrayl. According to Equation (2), transmittance and reflectance have an inverse relationship, the more ultrasonic waves transmitted into the container, the less echo energy reflected, and vice versa.

Ultrasonic waves can propagate in solids as longitudinal waves and transverse waves. Acoustoelastic effect means that in an isotropic solid medium, due to the effect of stress, the material has the characteristic of acoustoelasticity. That is, the ultrasonic wave velocity changes with the change of the stress state. But, ultrasonic waves can only propagate in the form of longitudinal wave in the liquid and gas medium, so the acoustoelastic effect is not considered.

### 2.2. Ultrasonic Near-Field and Far-Field Areas

**Figure 1:**
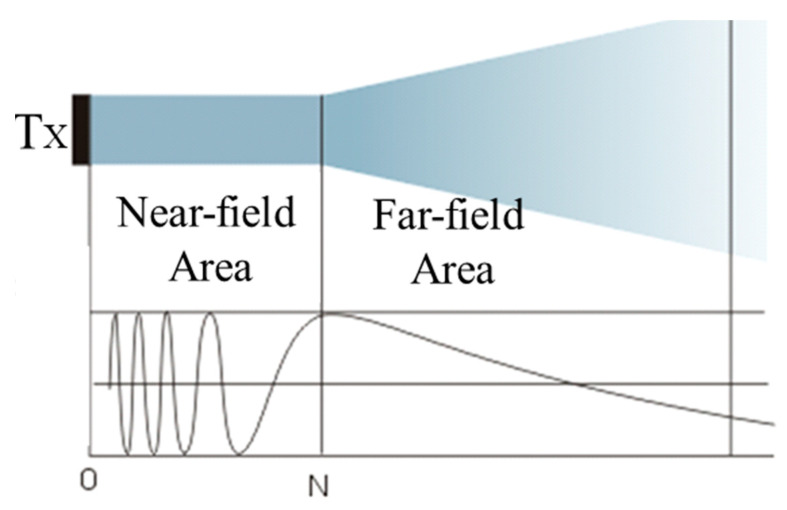
Ultrasonic sound field.

A beam of ultrasound emitted by an ultrasonic transducer includes both near-field and far-field areas [28]. The sound pressure near the wave source fluctuates sharply due to the interference of the wave and a series of sound pressure maximum and minimum appears, which is cylindrical in shape. At this time, the sound pressure is irregular, and the ultrasonic propagation is unstable [29]. The distance between the last sound pressure maximum value and the sound source is called the near field length, which is expressed by *N*, and the area within the *N* is called the near-field area. The region where the distance from the axis of the wave source to the wave source is greater than the length of the near-field region is divergent and is called the far-field region [30]. Its sound field diagram is shown in Figure 1. The ultrasonic near-field area can be calculated by Equation (3) [31]:

(3) $$N\approx \frac{D^{2}}{4\lambda }=\frac{A}{\pi \lambda }$$

where *D* is the ultrasonic sensor diameter, m, *A* is the sensor area, m^2^, and *λ* is the wavelength of ultrasonic wave propagation in the medium, which can be calculated using Equation (4):

(4) $$\lambda =\frac{c}{f}$$

where *c* is the wave velocity of ultrasonic wave propagation in the medium, m/s, and f is the ultrasonic frequency, Hz. Therefore, the near-field length of a beam of ultrasound is related to the diameter (area) of the piezoelectric plate and the speed and frequency of the ultrasound propagation in the medium. At a certain frequency and speed, the larger the diameter, the longer the near-field length.

The choice of buffer block material needs to consider several factors, of which the robustness, durability, and sensitivity are particularly important [21]. Puttmer et al. found that a low impedance material is more sensitive when its acoustic impedance is the same order of magnitude as the measured liquid [32]. The comparison of acoustic impedance of common buffer materials and water is shown in Table 1.

Polymers have a lower speed of sound than glass, ceramics, or metals, and even the thickness of the buffer block is small, and the delay effect is also good. Moreover, the polymer’s characteristic acoustic impedance is close to water, making it more sensitive [33]. Considering that the buffer block should have lower acoustic impedance and more regular acoustic characteristics, therefore, polymethyl methacrylate (PMMA) is selected as the buffer block in this paper. The characteristic acoustic impedance of PMMA is only 3.26 Mrayl, which is particularly suitable for measuring liquid acoustic characteristics using reflection technology.

### 2.3. Establishment of Experimental Platform

**Figure 2:**
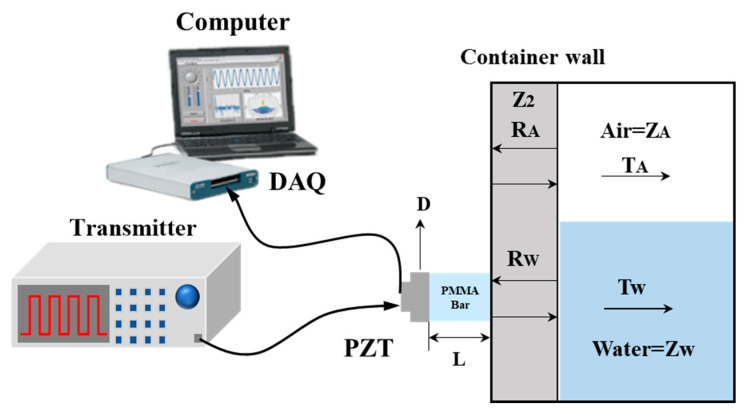
Designed structure of the liquid-level monitoring system. (PZT = Piezoelectric ceramic; D = Diameter; L = Length; Z_2_ = Aluminum alloy acoustic impedance; Z_A_ = Air acoustic impedance; Z_W_ = Water acoustic impedance R_A_ = Aluminum alloy–air interface reflection coefficient; T_A_ = Aluminum alloy–air interface transmission coefficient; R_W_ = Aluminum alloy–water interface reflection coefficient; T_W_ = Aluminum alloy–water interface transmission coefficient.).

According to the above theory, an external fixed-point liquid-level monitoring experimental platform is built. The square wave signal with a certain pulse width is amplified by the high-speed operational amplifier AD603 (Analog Devices, Inc., Norwood, LA, USA) and its peripheral circuits. Then, it drives the ultrasonic transducer to emit a group of continuous ultrasonic waves every 25 µs. The system only requires a double crystal probe, which is installed vertically on the outside of the container. A buffer block is placed between the probe and the container wall, and the gap is filled with an ultrasonic coupler to expel the air. When ultrasonic waves reach the interface between inner wall and internal medium, transmission and reflection phenomena occur. The reflected echo energy is received by the transducer, the data is collected by Data Acquisition (DAQ, BeijingXinChaoRenDa Technology Ltd., Beijing, China), and the waveform is finally displayed by the relevant software on the computer. The designed system is shown in Figure 2.

**Figure 3:**
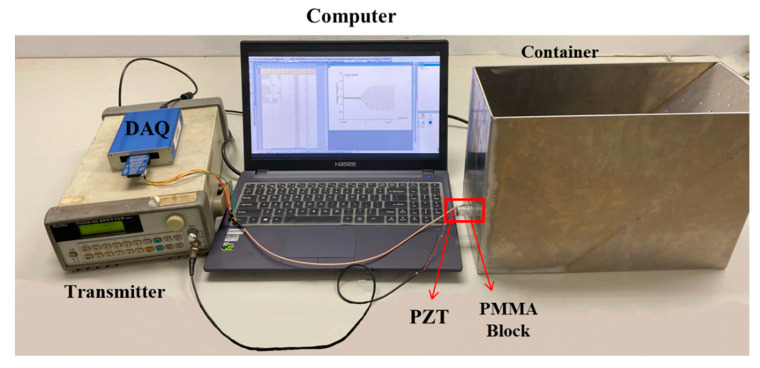
Photo of designed liquid-level monitoring system.

The tested container used in this experiment was made of aluminum alloy because gas tanks are mostly made of aluminum alloy in most aerospace and other applications. Its wall thickness is 3 mm. The internal measured medium is water and air. The material of the buffer block is PMMA, and the ultrasonic probe is piezoelectric ceramic (PZT). The experiment is carried out at a constant room temperature of 20 °C, so the change of sound velocity caused by temperature can be ignored. In addition, the sound velocity of PMMA varies approximately linearly with temperature and frequency, so the change of sound velocity caused by temperature will not affect the law presented by the evaluations in this paper, and the conclusions obtained through the experiment. Moreover, the variable in this experiment is the length of the buffer block PMMA. The density and material are constant, and its stress remains unchanged, which does not cause changes in the velocity of sound. The aluminum alloy container used in the experiment is not affected by stress. Therefore, we ignore the acoustoelastic effect in this paper. The device photo is shown in Figure 3. The relevant initial values of the experimental device and the measured liquid are shown in Table 2.

Substituting *Z*_2_, Z_W_, and Z_A_ in Table 2 into Equations (1) and (2) respectively, the reflection coefficient and transmission coefficient when the ultrasonic wave reaches the aluminum–water interface are: R_W_ = 83.10%, T_W_ = 16.90%, and at the aluminum alloy–air interface are: R_A_ = 99.99%, T_A_ = 0.01%. It can be concluded that the liquid medium has a higher transmission capacity than the gas medium. Thus, it is proven that the amplitude of ultrasonic echo received from the inner wall of the container when the liquid level is higher than the sensor should be significantly smaller than that when the liquid level is lower than the sensor.

**Figure 4:**
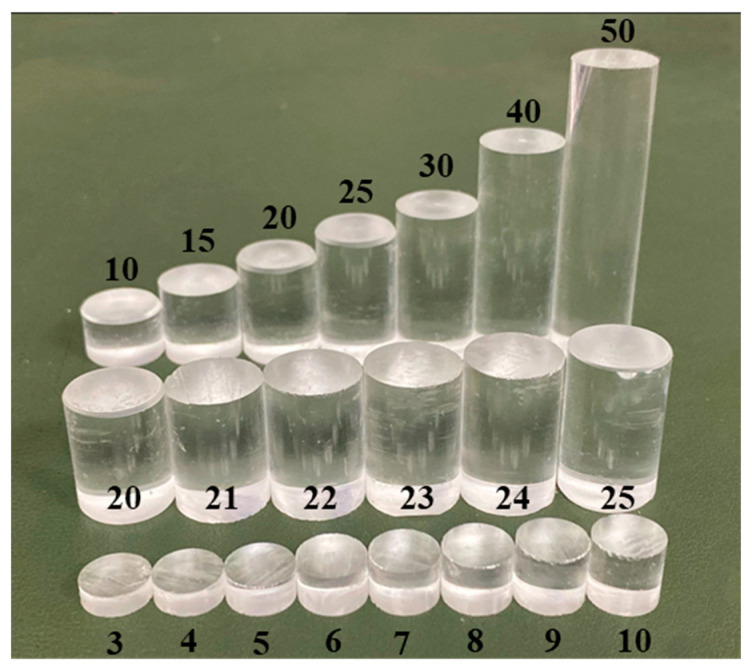
Photo of PMMA bars (mm).

Substituting *c* and *f*_0_ in Table 2 into Equation (4) can obtain the wavelength of ultrasonic waves propagating in PMMA, which is *λ* = $2.775\times 10^{-3}$ m. Substituting *λ*, *D*_1_, and *D*_2_ into Equation (3) gives *N*_1_ = 0.9 cm and *N_2_* = 2.03 cm. Therefore, in order to explore the relationship between the length of the near-field area and the ultrasonic echo energy, we customized some PMMA rods with different lengths and conducted experiments in groups. The physical picture of PMMA rods is shown in Figure 4. Its parameters are shown in Table 3.

## 3. Results and Discussion

Based on the above principles and devices, this paper conducted experiments in groups to explore the factors affecting the near-field length and the relationship between the length of the buffer blocks and the amplitudes of the ultrasonic echo.

### 3.1. Optimum Length Selection

**Figure 5:**
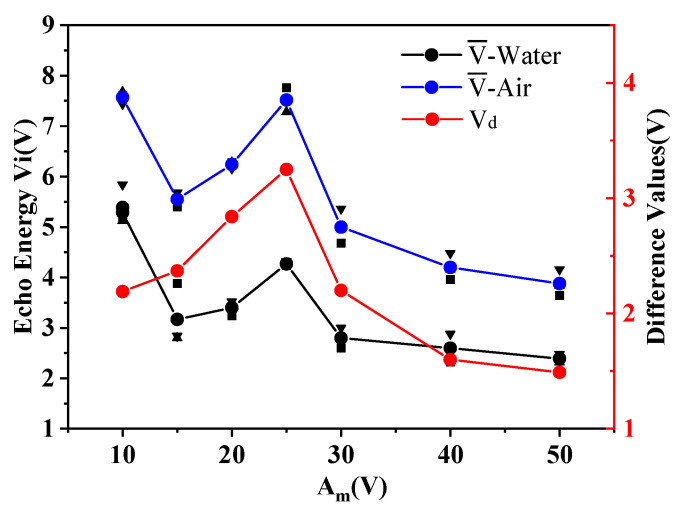
Results comparison diagram of the first group.

The ultrasonic probe with *D*_1_ = 15 mm, *f*_0_ = 1 MHz, *Am* = ±15 V in Table 2 and the buffer block with *D* = 15 mm and *L* = 10, 15, 20, 25, 30, 40, 50 mm in Table 3 were selected for the first group of experiments. The experiments were performed three times in each group. We recorded the amplitude of the echo received by the sensor and calculated the average values, $\bar{V}$, the average deviations, |∆E|, and the difference values, V_d_. The evaluations are shown in Table 4. Figure 5 shows the contents of Table 4.

**Figure 6:**
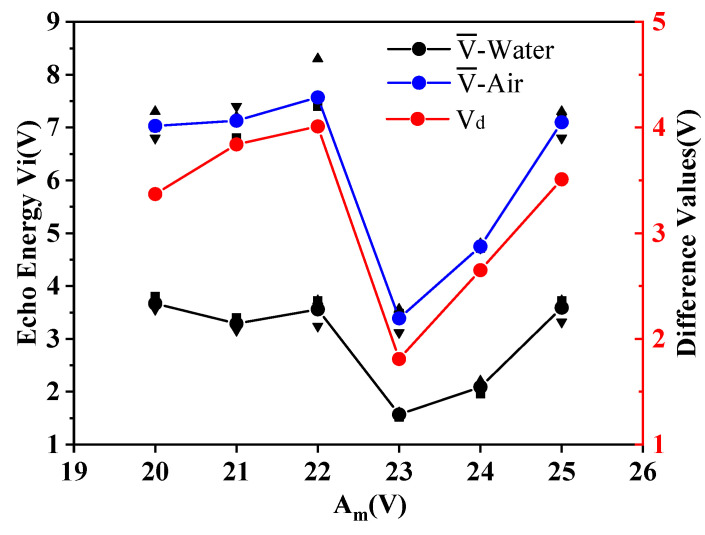
Results comparison diagram of the second group.

It can be seen from the experimental results in Table 4 and Figure 5 that under the condition of certain probe parameters, the experimental results are not good when the buffer block is too short or too long. When the length of the buffer block is 10 mm, the measured echo energy is the largest, but the energy difference between different mediums is small. When the length of the PMMA rod is 20 and 25 mm, the difference of echo voltage value between air and water inside the sensor is the largest, and the experimental effect is the most obvious. Therefore, PMMA rods with *D* = 15 mm and *L* = 20, 21, 22, 23, 24, and 25 mm in Table 3 were selected for the second group of experiments to find the optimal length of buffer block required in this experiment. The experiments were performed three times in each group. We recorded the amplitude of the echo received by the sensor and calculated the average values, $\bar{V}$, the average deviations, |∆E|, and the difference values, V_d_. The evaluations are shown in Table 5. Figure 6 shows the contents of Table 5.

It can be seen from the results that when the buffer block length is 22 mm, the echo difference between air and water reaches the maximum. At 23 mm, the difference drops to the lowest, and then the effect slowly gets better. Compared with the calculated length of the near-field area that *N*1 = 2.03 cm, the evaluations were basically consistent, and the average difference was less than 0.49 V. Therefore, this experiment has high reliability.

### 3.2. Minimum Length Selection

**Figure 7:**
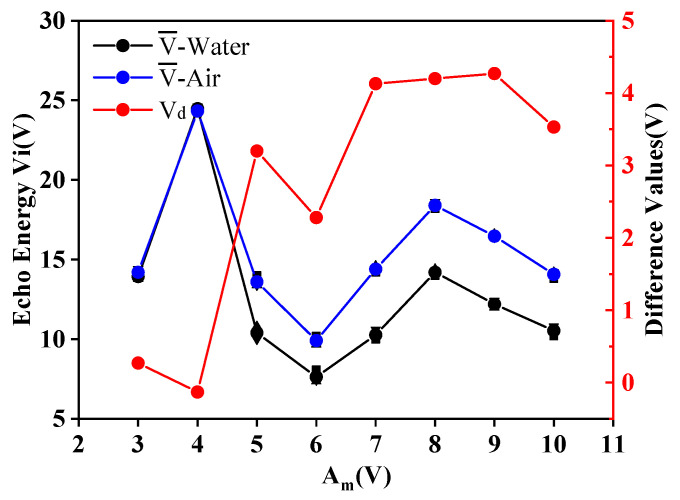
Results comparison diagram of the third group.

The minimum diameter of PZT available commercially is 10 mm. In order to find out the minimum size of ultrasonic probe that can be used in the liquid-level monitoring system in this paper, the ultrasonic probe with *D*2 = 10 mm, *f*_0_ = 1 MHz, *Am* = ±15 V in Table 2 and the buffer block with *D* = 15 mm and *L* = 3, 4, 5, 6, 7, 8, 9, and 10 mm in Table 3 were selected for the third group of experiments. The experiments were performed three times in each group. We recorded the amplitude of the echo received by the sensor and calculated the average values, $\bar{V}$, the average deviations, |∆E|, and the difference values, V_d_. The evaluations are shown in Table 6. Figure 7 shows the contents of Table 6.

According to Table 6 and Figure 7, when the buffer block length is 9 mm, the echo difference between air and water reaches the maximum, and the experimental effect is most ideal. Compared with the theoretical calculation, the length of the near-field area is *N_2_* = 9 mm, and the evaluations are consistent with the theoretical results. The average difference is less than 0.36 V. When the buffer block length is 5 mm, it can still effectively distinguish the medium type inside the sensor. However, when the length of the buffer block is less than 5 mm, the emitted ultrasonic wave propagates laterally along the container wall upon reaching the container wall, and generates several times more energy than the emitted ultrasonic wave. The data measured at this time are invalid. Therefore, the minimum size of the probe that can be used in this experiment is *D* = 10 mm in diameter and *L* = 5 mm in delay block length. The probe is 2 mm, so it is 7 mm in total.

**Figure 8:**
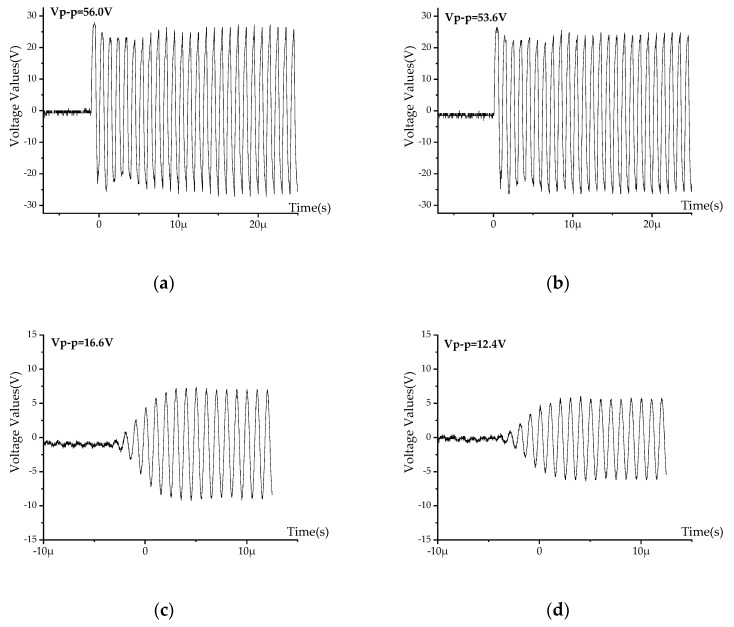
Echo images drawn by computer: (**a**) For air without buffer block, (**b**) for water without buffer block, (**c**) for air with 9 mm buffer block, and (**d**) for water with 9 mm buffer block.

The received echo signal is collected and displayed by the computer software. In the above experiment, the signal waveform obtained without buffer block is shown in Figure 8a, b. When a buffer block with a length of 9 mm is added between the probe and the container wall, the resulting signal waveform is shown in Figure 8c,d.

It can be seen from Figure 8 that without buffer block, the amplitudes of the echo signal cannot distinguish different internal mediums, and the waveforms do not have an envelope shape. It means that the emitted ultrasonic wave is in the near-field area, and the peak values are irregular, so the measurement results are invalid. Therefore, it is necessary to add a buffer block for transition when using ultrasonic probes for research. After adding the buffer block, the front part of the waveform is an envelope shape, followed by a sine shape with stable amplitudes. The echo energy in the air is greater than that in the water, with a difference of 4.27 V. Therefore, the platform built in this paper can be used to effectively monitor liquid level.

## 4. Conclusions

Based on the ultrasonic impedance method, we built a non-contact, fixed-point, liquid-level monitoring system. Then, we studied the relationship between the length of the near-field area and the ultrasonic echo energy. The evaluations show that under the situation of 20 °C in temperature, 1 MHz in ultrasonic probe frequency, and ±15 V in amplitude of the emitted ultrasonic wave, when the probe diameter is 15 mm, the optimal length of the buffer block is 22 mm. The maximum average difference of the results is 0.49 V. When ultrasonic probe is in its minimum size of 10 mm in diameter and 2 mm in thickness, the minimal length of the buffer block is 5 mm. The maximum average difference is 0.36 V and the evaluation precision is 1 mm. Our evaluations are consistent with the theory, which proves the reliability of the research. Our approach provides an effective solution to avoid the near-field area for all experiments based on ultrasonic. It provides a powerful basis for the selection and design of ultrasonic probes. The liquid-level monitoring system built in this paper has the advantages of convenient installation, high reliability, and high sensitivity. It can be applied to industrial and aerospace applications and has important practical significance. Moreover, the parameters such as the buffer block boundary shape can be further studied. Next, our team will research the influence of parameters such as tilt angle and width of buffer block on results in the oblique-incidence ultrasonic experiment, to design a complete set of ultrasonic probe structures.

## Figures and Tables

**Table 1:** Comparison of acoustic impedance of common buffer materials and water. PMMA: polymethyl methacrylate.

Materials	Material Types	Acoustic Impedance (Mrayl)	Reflectance (R)
Water	Liquid	1.48	100%
PMMA [34]	Polymer	3.26	37%
Quartz glass [35]	Glass	13.1	79.50%
Glass ceramics [22]	Glass	16.5	83.30%
Aluminum [36]	Metal	17.3	84%

**Table 2:** Experimental parameters and initial values.

Specification	Symbol	Initial Values
Container material	M_C_	Aluminum alloy 2219 (AL)
Wall thickness	h	3 mm
Transducer material	M_TX_	Piezoelectric ceramic (PZT)-5A
Buffer material	M_B_	PMMA
AL acoustic impedance	*Z*_2_	32 Mrayl
Water acoustic impedance	Z_W_	1.48 Mrayl
Air acoustic impedance	Z_A_	$4\times 10^{-4}$ Mrayl
PMMA acoustic impedance	Z_PMMA_	3.26 Mrayl
Ultrasound velocity	c	2775 m/s
Transducer diameter	D	10 mm, 15 mm
Working frequency	*f*_0_	MHz
Ultrasonic amplitude	Am	±15 V
Experimental temperature	T	20 °C

**Table 3:** Experimental PMMA parameters.

PMMA Diameters (mm)	PMMA Lengths (mm)
10	3, 4, 5, 6, 7, 8, 9, 10
15	10, 15, 20, 21, 22, 23, 24, 25, 30, 40, 50

**Table 4:** Evaluations of the first group.

L (mm)	Medium	V_1_ (V)	V_2_ (V)	V_3_ (V)	$\bar{V}$ (V)	|ΔE| (V)	V_d_ (V)
10	Water	5.20	5.12	5.84	5.39	0.30	2.19
	Air	7.60	7.68	7.44	7.57	0.09	
15	Water	3.88	2.80	2.84	3.17	0.47	2.37
	Air	5.40	5.56	5.68	5.55	0.10	
20	Water	3.24	3.44	3.52	3.40	0.11	2.84
	Air	6.28	6.28	6.16	6.24	0.05	
25	Water	4.24	4.24	4.32	4.27	0.04	3.25
	Air	7.76	7.28	7.52	7.52	0.16	
30	Water	2.60	2.80	3.00	2.80	0.13	2.20
	Air	4.68	4.96	5.36	5.00	0.24	
40	Water	2.32	2.60	2.88	2.60	0.19	1.60
	Air	3.96	4.16	4.48	4.20	0.19	
50	Water	2.36	2.32	2.48	2.39	0.06	1.49
	Air	3.64	3.84	4.16	3.88	0.19	

V_1_: The first measured voltage value; V_2_: The second measured voltage value; V_3_: The third measured voltage value; $\bar{V}$: The average values; |ΔE|: The average deviations; V_d_: The difference values.

**Table 5:** Evaluations of the second group.

L (mm)	Medium	V_1_ (V)	V_2_ (V)	V_3_ (V)	$\bar{V}$ (V)	|∆E| (V)	V_d_ (V)
20	Water	3.56	3.80	3.64	3.67	0.09	3.37
	Air	6.80	7.00	7.30	7.03	0.18	
21	Water	3.16	3.40	3.32	3.29	0.09	3.84
	Air	7.40	6.80	7.20	7.13	0.22	
22	Water	3.24	3.72	3.72	3.56	0.21	4.01
	Air	7.00	7.40	8.30	7.57	0.49	
23	Water	1.60	1.52	1.60	1.57	0.04	1.81
	Air	3.12	3.48	3.56	3.39	0.18	
24	Water	2.12	1.96	2.20	2.09	0.09	2.65
	Air	4.72	4.72	4.80	4.75	0.04	
25	Water	3.32	3.72	3.72	3.59	0.18	3.51
	Air	6.80	7.20	7.30	7.10	0.20	

**Table 6:** Evaluations of the third group.

L (mm)	Medium	V_1_ (V)	V_2_ (V)	V_3_ (V)	$\bar{V}$ (V)	|∆E| (V)	V_d_ (V)
10	Water	10.60	10.80	10.20	10.53	0.22	3.53
	Air	14.20	14.20	13.80	14.07	0.18	
9	Water	12.00	12.40	12.20	12.20	0.13	4.27
	Air	16.60	16.40	16.40	16.47	0.09	
8	Water	14.40	14.20	14.00	14.20	0.13	4.20
	Air	18.40	18.60	18.20	18.40	0.13	
7	Water	10.20	10.60	10.00	10.27	0.22	4.13
	Air	14.60	14.40	14.20	14.40	0.13	
6	Water	7.36	7.44	8.08	7.63	0.30	2.28
	Air	9.68	9.84	10.20	9.91	0.20	
5	Water	10.80	10.00	10.40	10.40	0.27	3.20
	Air	13.40	13.40	14.00	13.60	0.27	
4	Water	24.40	24.60	24.40	24.47	0.09	−0.13
	Air	24.40	24.40	24.20	24.33	0.09	
3	Water	13.80	14.00	14.00	13.93	0.09	0.27
	Air	14.00	14.40	14.20	14.20	0.13	
0	Water	53.60	54.00	53.00	53.53	0.36	2.27
	Air	56.00	55.60	55.80	55.80	0.13	
